# Cardiac coherence, self-regulation, autonomic stability, and psychosocial well-being

**DOI:** 10.3389/fpsyg.2014.01090

**Published:** 2014-09-29

**Authors:** Rollin McCraty, Maria A. Zayas

**Affiliations:** ^1^Institute of HeartMath, Boulder CreekCA, USA; ^2^Department of Psychology, Brenau UniversityGainesville, GA, USA

**Keywords:** coherence, trauma, heart rate variability, HRV, psychophysiological coherence, cardiac coherence, HeartMath, psychosocial well-being

## Abstract

The ability to alter one’s emotional responses is central to overall well-being and to effectively meeting the demands of life. One of the chief symptoms of events such as trauma, that overwhelm our capacities to successfully handle and adapt to them, is a shift in our internal baseline reference such that there ensues a repetitive activation of the traumatic event. This can result in high vigilance and over-sensitivity to environmental signals which are reflected in inappropriate emotional responses and autonomic nervous system dynamics. In this article we discuss the perspective that one’s ability to self-regulate the quality of feeling and emotion of one’s moment-to-moment experience is intimately tied to our physiology, and the reciprocal interactions among physiological, cognitive, and emotional systems. These interactions form the basis of information processing networks in which communication between systems occurs through the generation and transmission of rhythms and patterns of activity. Our discussion emphasizes the communication pathways between the heart and brain, as well as how these are related to cognitive and emotional function and self-regulatory capacity. We discuss the hypothesis that self-induced positive emotions increase the coherence in bodily processes, which is reflected in the pattern of the heart’s rhythm. This shift in the heart rhythm in turn plays an important role in facilitating higher cognitive functions, creating emotional stability and facilitating states of calm. Over time, this establishes a new inner-baseline reference, a type of implicit memory that organizes perception, feelings, and behavior. Without establishing a new baseline reference, people are at risk of getting “stuck” in familiar, yet unhealthy emotional and behavioral patterns and living their lives through the automatic filters of past familiar or traumatic experience.

## INTRODUCTION

The subjective experience of trauma is unique and varies according to the individual and the type of trauma. What does not vary is the fact that trauma often results in a devastating intrusion into a wished for life of peace, calm, and well-being along with a corresponding unexpected and undesired fragmented sense of self and of life in general. Most would agree that it is a lack of mental and emotional self-regulation that often characterizes stress, anxiety and overwhelm. For some, the lack of self-regulatory capacity is due to immaturity or skill acquisition while for others it can be due to trauma or impairment in the neural systems that underlie one’s ability to self-regulate. The degree of impairment in self-regulation that often characterizes trauma makes the possibility of a return to a state of wholeness and well-being appear as distant as an elusive memory. Nevertheless, most people have, at one time or another, likely known a balanced state that is typically characterized by feeling content, happy, in control and in sync within themselves and with others, and irrespective of circumstances and demographic factors, wish to regain this state, and feel good once again.

The quest to understand the mechanisms and dynamics of this sought-after state permeates the scientific and popular literature and gives rise to lines of thought and research that span a number of disciplines. In a broad sense, we call this experience of internal and external intra and interpersonal connectedness “coherence.” Through our research at the HeartMath Institute, we have come to identify a specific physiological state associated with optimal cognitive functioning and emotional stability and introduced the psychophysiological coherence model, briefly outlined below. This model is grounded in and consistent with research in the fields of neurocardiology, psychophysiology, and neuroscience (McCraty et al., 2009b).

The psychophysiological coherence model draws on dynamical systems theory. It emphasizes the importance of healthy physiological variability, feedback, inhibition, and reciprocal interactions among a hierarchy of nested neural systems that underlie a complex psychophysiological system for maintaining stability and adaptability to complex changing environments and social demands. Similar to the models proposed by Steve Porges (Porges, 2007) and Julian Thayer (Thayer et al., 2009), the coherence model also suggests that the amount of heart rate variability (HRV) that is mediated by efferent vagal fibers, reflects self-regulatory capacity and that low age-adjusted HRV indexes a low functional status of the system.

It is our perspective that each of these models introduces important aspects of the neural systems involved in emotional experience and the self-regulation of emotions and behaviors, and together they more fully describe the evolution, anatomy, and functions of these psychophysiological control systems. As these models are discussed in detail elsewhere and in other articles in this issue, they will not be discussed here.

A growing number of studies have linked vagally mediated HRV to self-regulatory capacity (Segerstrom and Nes, 2007; Geisler and Kubiak, 2009; Reynard et al., 2011), emotion regulation (Appelhans and Luecken, 2006; Geisler et al., 2010), social interactions (Smith et al., 2011; Geisler et al., 2013), one’s sense of coherence (Miller et al., 1960) and personality character traits of Self-Directedness (Zohar et al., 2013) and coping styles (Ramaekers et al., 1998).

We use the terms cardiac coherence and physiological coherence interchangeably to describe the measurement of the order, stability, and harmony in the oscillatory outputs of the regulatory systems during any period of time. For example, resting HRV data obtained from a population of returning soldiers with a diagnosis of post-traumatic stress disorder (PTSD) found that those with a diagnosis of PTSD had both lower levels of HRV and lower levels of coherence than control subjects without PTSD (Ginsberg et al., 2010). In a study of the effects of playing violent and nonviolent video games, it was found that when playing violent video games, the players had lower cardiac coherence levels and higher aggression levels than did nonviolent game players and that higher levels of coherence was negatively related to aggression (Hasan et al., 2013).

The psychophysiological coherence model predicted that different emotions are reflected in state-specific patterns in the heart’s rhythms (HR; McCraty et al., 2009b) independent of the amount of HRV, although state-specific changes in the amount of HRV are of course also important. We can usually identify patterns associated with anxiety versus frustration or anger, for example, by looking at the HRV waveforms. Recent independent work has verified this by demonstrating a 75% accuracy in detection of discrete emotional states from the HRV signal using a neural network approach for pattern recognition (Leon et al., 2010). Several studies in healthy subjects, which help inform the model, showed that during the experience of positive emotions a sine wave-like pattern naturally emerges in the HR without any conscious changes in breathing (McCraty et al., 1995; Tiller et al., 1996). This is likely due to more organized outputs of the subcortical structures involved in processing emotional information described by Pribram and Melges (1969), Porges (2007), and Thayer et al. (2009) in their Central Autonomic Network model in which the subcortical structures influence the oscillatory output of cardiorespiratory centers in the brain stem. Thus, the term psychophysiological coherence is used in the context of when more coherent heart rhythms naturally emerge due to positive experience, or through the self-activation of positive emotions. This is associated with a different subjective inner state that is achieved through techniques such as paced breathing that increase cross-coherence between breathing and heart rhythms via brainstem centers in the medulla, but do not necessarily shift the activity in higher level sub-cortical structures that appear to mediate the structure of different patterns in the HRV waveforms and the increased or decreased coherence related to emotional states (McCraty et al., 2009b). An important aspect of the coherence model (not the specific measure of HRV coherence) is focused on specific approaches to increase people’s ability to self-regulate. In this context the HRV coherence measure is intended to be used in the context of facilitating skill acquisition of self-regulation practices that lead to measurable increases in HRV coherence.

The coherence model informed the development of a number of mental and emotional self-regulation techniques, most of which are designed to be used in the moment one is emotionally triggered or is experiencing stress, or to better prepare for upcoming challenging events (Childre and Martin, 1999). The use of these techniques typically shifts the user’s physiology into a more coherent and balanced functional state which is reflected in the patterns of the heart’s rhythm.

We have found that regular practice of these intentionally simple self-regulation techniques, most of which instruct users to place their attention in the center of the chest and then self-activate a feeling of calmness or a positive emotion, can lead to lasting increases in participants’ ability to self-regulate and maintain their composure. Use of the techniques also leads to a state-specific increase in HRV and vagal activity (vagal tone; McCraty et al., 1995; Tiller et al., 1996) and over time can lead to sustained increases in HRV. In a study of high school students who practiced the self-regulation techniques over a three month period, their resting HRV was significantly increased and the pattern of the HRV was significantly more coherent. These improvements in resting HRV coherence were significantly correlated with increased test scores and improved behaviors, suggesting that the practice of the self-regulation skills induces a more coherent heart rhythm, reinforcing the association in the sub-cortical regulatory systems involved in a match/mismatch process between more coherent and stable rhythms in cardiovascular afferent neuronal traffic and feelings we perceive as positive (Bradley et al., 2010).

By reinforcing this natural coupling in the sub-cortical regulatory systems, the self-activation of a positive feeling can automatically initiate an increase in cardiac coherence, while at the same time, a physiological shift resulting from heart-focused breathing can help facilitate the experience of a positive emotion.

An important aspect of the coherence model is the inclusion of cardiovascular afferent neuronal inputs on sub-cortical and cortical structures, which can have significant influences on cognitive resources and emotions. Formally introduced as the “Heart Rhythm Coherence Hypothesis,” we proposed that information is conveyed in the patterns of the HR which reflects current emotional states, and that the patterns of afferent neural input (coherence and incoherence) to the brain affect emotional experience, and modulate cortical function and self-regulatory capacity over macroscopic time scales. Furthermore, we proposed that intentional activation of positive emotions plays an important role in increasing cardiac coherence and increasing self-regulatory capacity (McCraty et al., 2009b). Our findings expand on a large body of research on the benefits of positive emotional states on physical, mental, and emotional health (Isen, 1999; Fredrickson, 2001, 2002; Fredrickson and Joiner, 2002; Fredrickson et al., 2003; Wichers et al., 2007).

This paper provides a brief summary of the psychophysiological coherence model and its implications for improving mental and emotional health and self-regulation. A detailed discussion on the nature of coherence can be found in two seminal articles (McCraty et al., 2009b; McCraty and Childre, 2010). We also provide a brief review of some of the studies that have investigated the coherence-based approach to increasing self-regulatory capacity, physical health, cognitive function, and psychosocial well-being in various populations. Trauma-specific applications with respect to the mechanisms whereby the impact of trauma may be attenuated are considered, with special emphasis on the importance of shifting the internal baseline reference that can be considered a type of implicit memory held in the neural architecture that helps organize perception, feelings and behavior. Of particular relevance to this discussion, is an understanding of the continuum of functions related to mental and emotional self-regulation, and approaches for shifting autonomic nervous system (ANS) activity to one which is increasingly balanced and coherent. As the system becomes more coherent, it becomes possible to reestablish wholeness to the fragmented experience of self and of one’s life that is common in individuals who have undergone traumatic events.

## PSYCHOPHYSIOLOGICAL COHERENCE OVERVIEW

The coherence model postulates that: (1) The functional status of the underlying psychophysiological systems determines the range of one’s ability to adapt to challenges, self-regulate and engage in harmonious social relationships. Healthy physiological variability, feedback systems, and inhibition are key elements of the complex system for maintaining stability and capacity to appropriately respond to and adapt to changing environments and social demands. (2) The oscillatory activity in the HR reflects the status of a network of flexible relationships among dynamic interconnected neural structures in the central and ANSs. (3) State-specific emotions are reflected in the patterns of the HR independent of changes in the amount of HRV. (4) Sub-cortical structures constantly compare information from internal and external sensory systems via a match/mismatch process that evaluates current inputs against past experience to appraise the environment for risk or comfort and safety. (5) Physiological or cardiac coherence is reflected in a more ordered sine wave-like heart rhythm pattern associated with increased vagally mediated HRV, entrainment between respiratory, blood pressure and heart rhythms, and increased synchronization between various rhythms in the EEG and the cardiac cycle. (6) Vagally mediated efferent HRV provides an index of the cognitive and emotional resources needed for efficient functioning in challenging environments in which delayed responding and behavioral inhibition are critical. (7) Information is encoded in the time between intervals (action potentials, pulsatile release of hormones, etc.). The information contained in the inter-beat-intervals in the heart’s activity is communicated across multiple systems and helps synchronize the system as a whole. (8) Patterns in the activity of cardiovascular afferent neuronal traffic can significantly influence cognitive performance, emotional experience, and self-regulatory capacity via inputs to the thalamus, amygdala, and other sub-cortical structures. (9) Increased “rate of change” in cardiac sensory neurons (transducing BP, rhythm, etc.) during coherent states increases vagal afferent neuronal traffic which inhibits thalamic pain pathways at the level of the spinal cord. (10) Self-induced positive emotions can shift the psychophysiological systems into a more globally coherent and harmonious order associated with improved performance and overall well-being.

## PSYCHOPHYSIOLOGICAL COHERENCE AND WELL-BEING

As discussed earlier, this model was used to develop simple techniques that allow people to quickly self-induce a physiological shift to a more coherent state which takes advantage of the concurrent change in afferent neuronal input to the brain which is associated with increased self-regulatory capacity and thus ability to more successfully handle the demands and challenges of life with more ease and composure. Consequently, there is a greater experience of connectedness, harmony, balance and physical, emotional, and psychosocial well-being.

Outcome studies conducted in laboratory, clinical, educational, and organizational settings with diverse populations have shown sustained reductions in stress and improvements in many dimensions of health, well-being, and performance. For example, a study of middle school students who had a diagnosis of attention deficit hyperactivity disorder (ADHD) demonstrated that the students had a wide range of significant improvements in cognitive functions such as short and long-term memory, ability to focus, and significant improvements in behaviors, both at home and in school (Lloyd et al., 2010). A study of 41 fighter pilots engaging in flight simulator tasks found a significant correlation between higher levels of performance and heart rhythm coherence as well as lower levels of frustration (Li et al., 2013).

Other studies have shown the use of these self-regulation techniques increases parasympathetic activity (HF power) (Tiller et al., 1996) and results in significant reductions in cortisol and increases in DHEA over a 30 day period (McCraty et al., 1998). Studies also show significantly lowered blood pressure and stress measures in a population with a diagnosis of hypertension (McCraty et al., 2003). A study of hypertensive patients showed that those who used the techniques to increase HRV coherence had significantly greater reduction in mean arterial pressure than those who were taking hypertensive medications and using relaxation techniques (Alabdulgader, 2012). A controlled study of pastors found significant improvements in stress and well-being measures with an overall decrease in health care costs of $585 per participant, while the control group had a 9% increase in health care costs. The largest reduction in costs was related to reductions in medications for hypertension (Bedell, 2010). A study of patients with congestive heart failure also showed significant improvements in functional capacity and reduced depression as compared to a control group (Luskin et al., 1999).

While overall health and wellness benefits have been associated with increased coherence, there is also evidence related more specifically to trauma and high stress populations. A study at the Columbia, South Carolina VA hospital of recently returning soldiers from Iraq who were diagnosed with PTSD, found that relatively brief periods of cardiac coherence training combined with practicing the Quick Coherence technique resulted in significant improvements in the ability to self-regulate along with significant improvements in a wide range of cognitive functions, which correlated with increased cardiac coherence (Ginsberg et al., 2010). In a study of returning veterans with chronic pain, the treatment group showed marked and statistically significant increases in coherence (191%) along with significant reductions in pain ratings (36%), stress perception (16%), negative emotions (49%), and physical activity limitations (42%) (Berry et al., 2014). In a study of patients with severe brain injury, it was found that the emotion self-regulation training resulted in significantly higher coherence ratios and higher attention scores. Additionally, the families’ ratings of participants’ emotional control correlated with improved HRV indices (Kim et al., 2013b).

A study of correctional officers reporting high work stress showed significant reductions in systolic and diastolic BP, total cholesterol, fasting glucose, overall stress, anger, fatigue, and hostility (McCraty et al., 2009a,b).

Similar results were obtained in several studies with police officers where it was found that the officers’ capacity to recognize and self-regulate their responses to stressors in both work and personal contexts was significantly improved. Officers experienced reductions in stress, negative emotions, depression, and increased peacefulness and vitality as compared to a control group. In the qualitative aspect of the study, officers reported improved family relations, better communication and cooperation at work (McCraty and Atkinson, 2012; Weltman et al., 2014).

## TRAUMA

Just as the experience of coherence is one of harmonious synchronization and flow, the experience of trauma is often one of disconnectedness, alienation, and dysregulation (Berntsen et al., 2003; Emerson and Hopper, 2011). Neurologist, Robert Scaer (Scaer, 2012) examined the nature of trauma from a neuroscience perspective and paints a picture of a depersonalized reality in which the trauma experience is frozen in time, where procedural and emotion-linked declarative memories become encapsulated elements which are repeatedly brought into one’s current consciousness by internal or external cues associated with the traumatic event. These recurring intrusions seem inescapable and threatening and are beyond one’s capacity to control them. Being subject to these temporal alterations removes a person from the present moment, resulting in a fragmented experience of life devoid of a sense of control, wholeness or meaningful connections with others. Scaer (2012) also explored the nature of bidirectional interactions between the various parts of the brain during these episodes and highlighted the deactivation of the frontal cortex and the hyper-activity of the limbic system, in particular, the amygdala. Given the role of the frontal cortex in self-regulation, strategic thinking, decision-making, empathy, and relatedness, the necessity for facilitating cortical function and down-regulating activity in the amygdala in order to achieve optimal personal function and psychosocial well-being becomes clear.

While the abundant literature examining the spectrum of trauma has produced numerous conceptualizations of the etiology of trauma, the various correlates of trauma in psychological and social function, and the recommended treatment approaches to address the complex aspects and impact of trauma, there is consensus in the research that trauma is characterized by a disruption in one’s ability to respond appropriately to a perceived threat (Levine, 2008) and that physiological factors underlie cognitive, behavioral, and social function (Levine, 2010). Neurological correlates of trauma are well-documented. Emerging research in the field of neurocardiology and psychophysiology provides an expanded understanding of the role of the physiological aspect of trauma, particularly with respect to self-regulation, HRV, and how these relate to restoring optimal function along an integrated continuum (Porges, 2007; Thayer et al., 2009).

Of primary importance in the process of facilitating a return to optimal function is the realization that trauma is associated with emotional dysregulation pursuant to ongoing activation by trauma cues and a corresponding inability to return to physiological homeostasis (Norte et al., 2013). Cardiac function has been clearly implicated in this mechanism, particularly with respect to HRV (Frustaci et al., 2010). For example, it has been shown that elevated psychophysiological baseline scores and heightened physiological reactivity to trauma-related cues are typical features of PTSD which can be objectively measured through cardiac parameters (Keane et al., 1998). A recent twin study (Shah et al., 2013) found a significant relationship between autonomic function and PTSD, independent of other potentially confounding variables such as genetic, familial, and socio-demographics. After adjusting for cardiovascular risks, depression and history of substance abuse, the researchers stated: “…we were able to demonstrate a dose response relationship between PTSD symptom severity and HRV. In contrast, we found a mostly null association between remitted PTSD and autonomic function, suggesting possible reversibility of autonomic dysregulation after PTSD symptom resolution (p. 1106).”

A recent DoD-funded study examined pre-deployment HRV as a predictor of post-deployment PTSD symptoms and compared a HRV coherence biofeedback resilience training to a no-additional training control group in a population of Army National Guard soldiers. Preliminary results demonstrated that lower pre-deployment HRV (SDNN) was a significant predictor of post-deployment PTSD symptoms. The HRV biofeedback resilience training group resulted in lower PTSD symptom severity for soldiers 26 years of age and older (personal communication with Jeff Pyne, Center for Mental Health Outcomes Research Central Arkansas Veterans Healthcare System). Understanding these relationships between physiological baselines as they relate to HRV is essential to developing effective treatment modalities for individuals experiencing trauma. In order to explore this topic at a deeper level, it becomes important to first examine the dynamics of self-regulation in the context of cardiac coherence.

## EMOTIONS AND HEART RHYTHM PATTERNS

**FIGURE 1:**
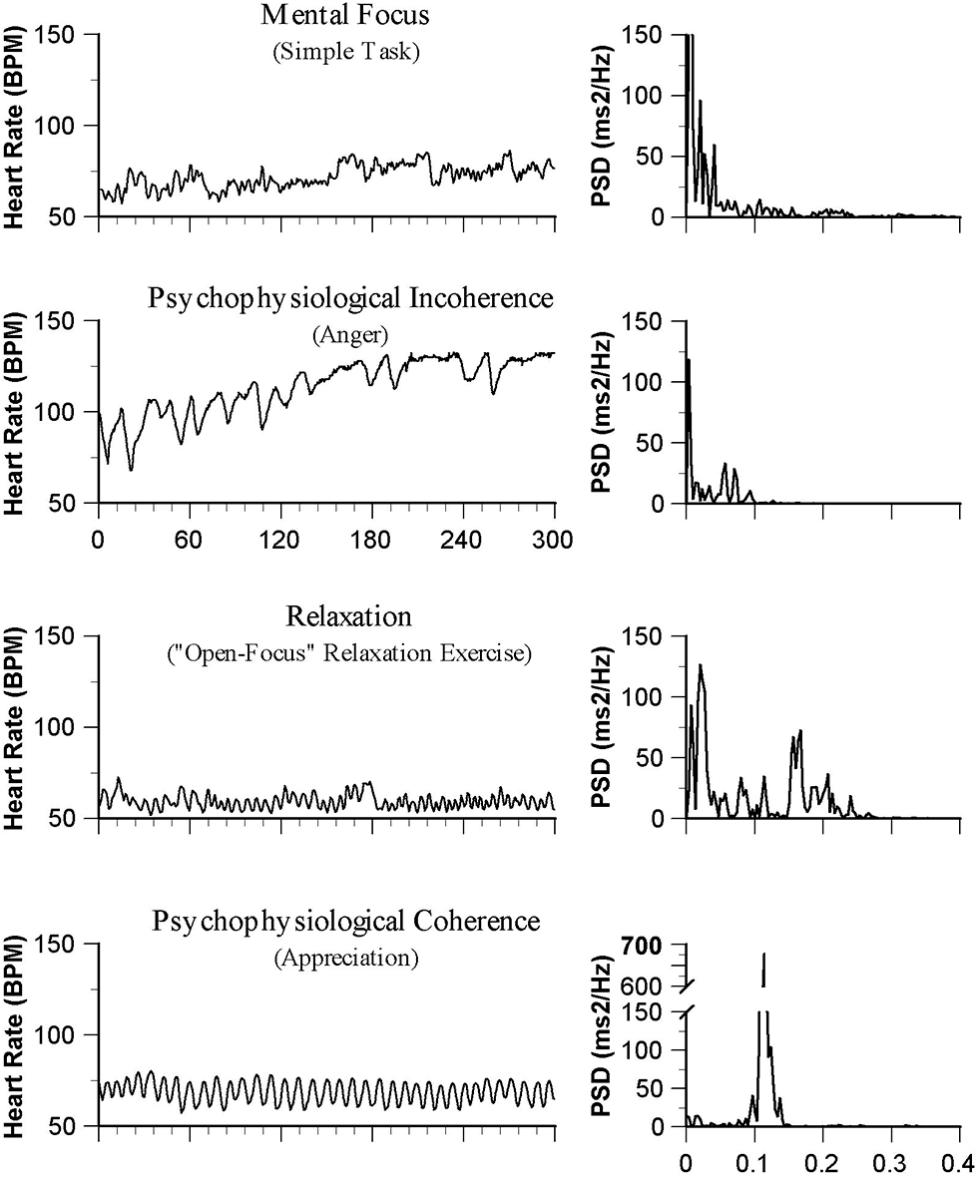
**Emotions and heart rhythm patterns.** The heart rate tachograms on the left side show patterns in of the HRV waveforms typically observed in differing psychological states. The power spectral density (PSD) analysis of the HRV rhythms for each is shown on the left.

One of the most salient findings which is of particular relevance to this discussion relates to the association between the quality of emotional experience and the patterns reflected in HRV waveforms, including coherence. The nature of the emotional experience appears to be related to the level of coherence of the heart rhythm pattern (McCraty et al., 1995, 2009b). **Figure 1** illustrates that emotions typically thought of as positive, such as appreciation and compassion, are related to a more coherent heart rhythm pattern; whereas, emotions that are typically thought of as negative are related to more incoherent pattern, suggesting that positive emotions may have a renewing physiological effect and negative emotions may have a depleting physiological effect.

Although heart rate changes often occur with emotional state changes, we have found that it is more typically the patterns reflected in the heart’s rhythm that change in a state-specific manner, especially during emotions that do not evoke large ANS activations or inhibitions of parasympathetic outflow. “These changes in rhythmic patterns are independent of heart rate; that is, one can have a coherent or incoherent pattern at higher or lower heart rates.” “Thus, it is the pattern of the rhythm (the ordering of changes in rate over time), rather than the heart rate (at any point in time) that reflects the more subtle ANS and emotional dynamics as well as physiological synchronization” (McCraty and Childre, 2010; p. 12). From a physiological perspective, a coherent heart rhythm is different than the heart rhythm that occurs during the relaxation response, which is associated with a reduced heart rate, but not necessarily a more coherent rhythm.

Physiological coherence is reflected in more ordered and sine wave-like HRV patterns at a frequency of around 0.1 Hz (10 seconds rhythm). A coherent rhythm can be defined as “a relatively harmonic (sine wave-like) signal with a very narrow, high-amplitude peak in the LF region of the HRV power spectrum with no major peaks in the VLF or HF regions. Coherence is assessed by identifying the maximum peak in the 0.04–0.26 Hz range of the HRV power spectrum, calculating the integral in a window 0.03 Hz wide centered on the highest peak in that region, and then calculating the total power of the entire spectrum. The coherence ratio is formulated as: [Peak Power/(Total Power - Peak Power)]” (McCraty and Childre, 2010; p.14).

## HEART–BRAIN COMMUNICATION

The coherence hypothesis suggests that the coherent flow of information within and between the physiological systems and processes in the central and ANS and body plays an important role in determining the quality of the feelings and emotions one experiences.

Heart rate variability analysis, therefore, becomes an important tool that provides a window into the activity occurring between the heart and brain, as well as within regulatory centers in the brain. HRV is generated largely by the interaction between the heart and brain via the neural signals flowing through the afferent (ascending) and efferent (descending) neural pathways of the sympathetic and parasympathetic branches of the ANS (Malik and Camm, 1995; McCraty et al., 1995; Kamath et al., 2013).

Specific HRV variables are used to assess the beat-to-beat changes in heart rate associated with rhythms generated by different physiological mechanisms. The various HRV measures can be used to gain insights into the complex interactions between the central nervous system, the ANS and the heart (McCraty et al., 2009b). An appropriate level of physiological variability in the regulatory systems reflects an organism’s flexibility and ability to coherently adapt to stress and challenges (Segerstrom and Nes, 2007). The overall amount of HRV one has, which is best assessed over a 24 hour period, is related to age, with older people having lower levels than younger people (Umetani et al., 1998). Low age-adjusted HRV, especially in the VLF and ULF bands, has been shown to be associated with increased health risk in a wide range of clinical conditions and all-cause mortality (Saul et al., 1988; Arrone et al., 1997; Levy et al., 2002; Lindmark et al., 2003, 2005, 2006). HRV, especially the HF band, provides an index of psychological resiliency, behavioral flexibility and one’s capacity to adapt to changing social demands (Beauchaine, 2001). In addition, Thayer and Lane’s model describing a dynamic system of neural structures that they call the central autonomic network links cognitive performance with autonomic regulation and HRV. It has been shown in a series of studies that resting levels of HRV are predictive of individual differences in performance on tasks requiring utilization of the prefrontal structures underlying executive functions (Thayer et al., 2009).

## CARDIOVASCULAR AFFERENT NEURONS

**FIGURE 2:**
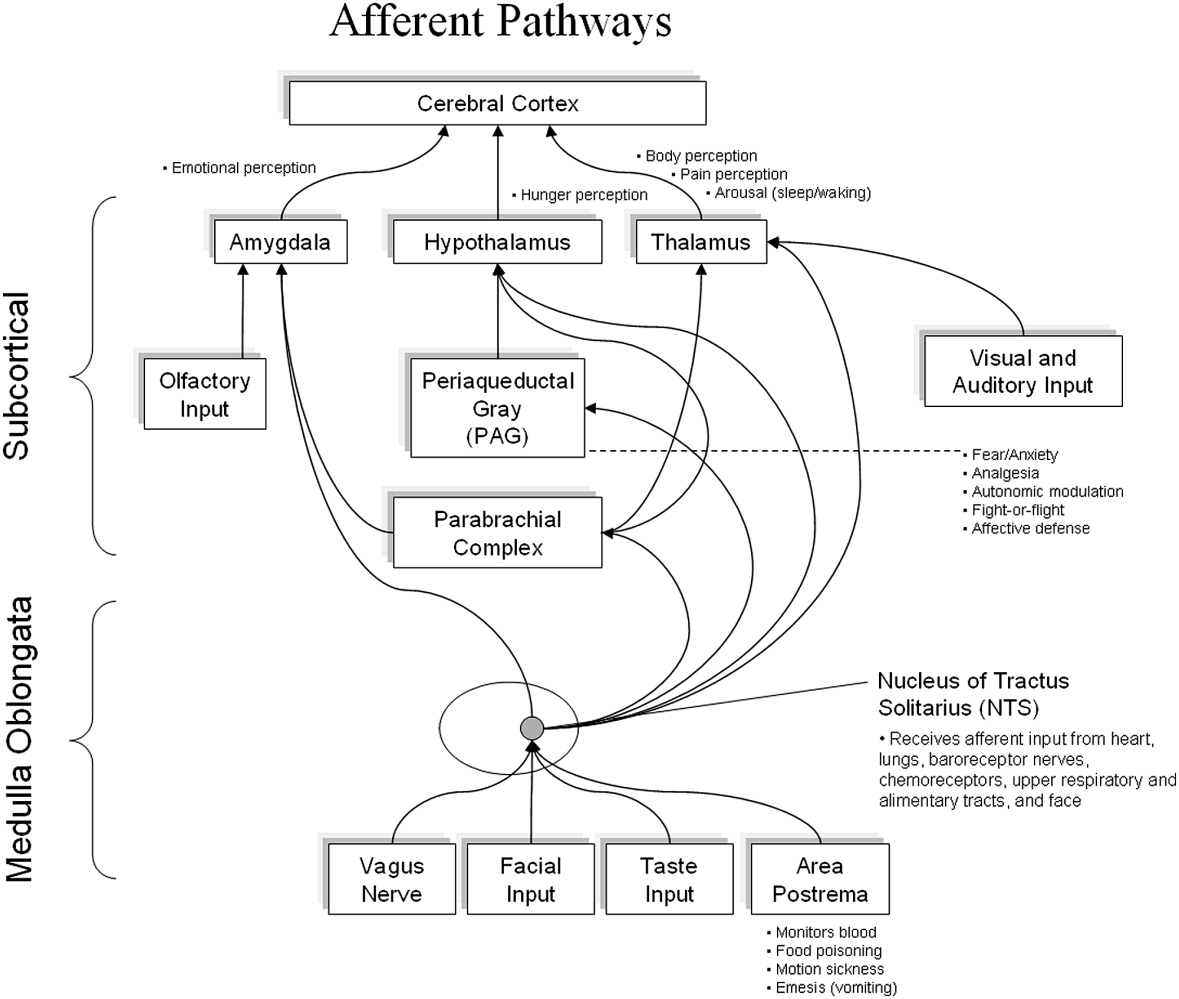
**It shows the major afferent inputs from the body to the Dorsal Vagal Complex.** Afferent pathways then connect directly to the amygdala, hypothalamus, and thalamus, etc. There is emerging evidence of a direct connection from the NTS to the frontal cortex.

Porges (2007) also points out that there has been a bias in most text books to focus only on the efferent pathways in the ANS and neglect the role of the afferent neurons as part of the dynamic regulatory system. Therefore, it is less commonly understood that 85–90% of the fibers in the vagus nerve are afferent (Cameron, 2002) and that cardiovascular related afferent neural traffic significantly affects activity in the majority of higher brain centers, as well as cognitive processes and emotional experience (McCraty et al., 2009b). As shown in **Figure 2**, cardiovascular afferents have connections to numerous brain centers including the thalamus, hypothalamus, and amygdala.

Lacey (1967) and Lacey and Lacey (1970, 1974) were the first to demonstrate a causal relationship between perceptual and sensory-motor performance and cardiovascular afferent neuronal activity. Wölk and Velden (1987) updated their hypothesis after demonstrating that cognitive performance was actually modulated at a rhythm around 10 Hz. In essence, they demonstrated that afferent neuronal activity modulated cortical function by inhibiting or facilitating synchronization of global cortical activity that was mediated by afferent pathways to the thalamus (Velden and Wölk, 1987; Wölk and Velden, 1987, 1989). Importantly, they found it was the *pattern* and *stability* of the rhythm in the cardiovascular afferent neuronal input as opposed to the rate of neural bursts that were important.

Numerous anatomical and neural recording studies in the field of neurocardiology have since shown that the neural communication between the heart and brain is far more complex than has been traditionally understood (Armour and Ardell, 1994). This body of work found that patterns of complex afferent information is continuously sent to the brain (not just within the cardiac cycle) and is related to mechanical and chemical factors over time scales ranging from milliseconds to minutes (Armour and Kember, 2004).

Thus, the heart rhythm coherence hypothesis “postulates that the pattern and degree of stability in the beat-to-beat changes in heart rate encodes information over macroscopic time scales which can influence cognitive performance and emotional experience” (McCraty et al., 2009b; p. 16). Several studies have established a relationship between interventions that increase the coherence in the HR and significant improvements in cognitive performance (Bradley et al., 2010; Ginsberg et al., 2010; Lloyd et al., 2010). In a study utilizing an odd-ball audio discrimination task with reaction times and error rates as measures of cognitive performance, participants used a technique to induce either a coherence state or a relaxation state for 5 min prior to the experimental protocol. In the coherence group, there was a carry-over effect on subsequent performance as compared to the relaxation group, and there was a significant correlation between pre-task cardiac coherence and performance across all participants. Additionally, the significant performance improvement was six times larger than that observed due to afferent neuronal effects that occur within a single cardiac cycle (McCraty et al., 2009b).

## VAGAL AFFERENT NERVE TRAFFIC

One of the properties of sensory neurons (baroreceptors) is that they are most responsive to increases in rate of change in the function they are tuned to detect (heart rate, pressure, etc.) (Armour and Ardell, 1994). During periods of increased cardiac coherence, there is typically an increased range of variability in both blood pressure and heart rate, which is detected as increases in the rate of change by the sensory neurons, resulting in increased firing rates which increases vagal afferent traffic. There is also a more ordered pattern of activity. A recent study using heartbeat evoked potential showed that using paced breathing at the coherence rhythm increased both the range of HRV and the coherence in the rhythms as expected, and also increased the N200 amplitude potential in the EEG heartbeat evoked potentials indicative of increased afferent input (MacKinnon et al., 2013). Foreman (1989, 1994, 1997) has done a series of anatomical and stimulation studies that have shown the thalamic pain pathways in the spinal cord are inhibited by increases over the normal intrinsic levels of vagal afferent nerve traffic. It has also been demonstrated that vagal afferent nerve stimulation reduces migraine and cluster headaches (Mauskop, 2005). Vagal nerve stimulation has also been shown to improve cognitive processing and memory (Hassert et al., 2004). There has also been a growing number of studies in recent years using afferent vagal stimulation in a wide range of clinical disorders such as epilepsy, obesity, depression, anxiety, autism, alcohol addiction, mood disorders, multiple sclerosis, and traumatic brain injury (Kosel and Schlaepfer, 2003; Groves and Brown, 2005).

Importantly, Lehrer et al. (2006) has shown that regular practice of HRV biofeedback results in lasting improvements in baroreflex gain independent of cardiovascular and respiratory effects, demonstrating neuroplasticity within the baroreflex system, likely in the intrinsic cardiac nervous system. Thus, repeated sessions of heart coherence can reset the baroreflex system gain resulting in increased afferent nerve activity noninvasively.

## ESTABLISHING A NEW BASELINE

In order to understand how increased cardiac coherence facilitates self-regulation and helps reset the regulatory systems in cases of trauma, it becomes necessary to examine the emerging perspective in neuroscience that emotions reflect complex somatic states (Cameron, 2002; Damasio, 2003) that become “set points” in the neural architecture which act as a type of implicit memory, or baseline reference (Pribram and Melges, 1969).

Pribram’s (1970) theory postulates that emotional information is carried by various internal rhythms, most notably from the heart and facial expressions, in the form of low frequency oscillations produced by these systems. He further proposes that higher frequency oscillations (EEG) relate to the process of making perceptual interpretations of sensory stimuli in the environment. Specifically, he considers the brain’s information monitoring role to be a central component of this process. As the brain monitors these inputs, neural patterns are established in nested feedback loops in the neural architecture. This implicit memory functions as a baseline against which we assess all sensory input (Pribram, 1970).

In other words, we establish physiological and behavioral set points or default patterns that, once established, the brain and nervous system strive to maintain. Although more complex, this is analogous to setting the temperature to a specific setting on a thermostat that the heating system works to maintain. It is important to note that the default patterns that are established are adaptive and while appropriate in one context, may not be healthy or optimal in another.

Once a stable pattern is formed and established in memory, all sensory input to the brain from both the internal and external sensory systems is compared to the reference patterns and programs. When the current inputs match the baseline pattern, the brain recognizes them as familiar, which we experience as comfortable and safe. It is important to note that this same process occurs even if the reference pattern is one that is associated with anxiety, chaos, confusion, overwhelm, etc. It becomes comfortable because it is familiar.

In order to maintain stability and feelings of safety and comfort, we must be able to maintain a match between our current experience or “reality” and one of our previously established neural programs (Miller et al., 1960). When we encounter a new experience or challenge, there can be a mismatch between the input patterns of the new experience and the lack of a familiar reference. Depending on the degree of mismatch, it requires either an internal adjustment (self-regulation) or an outward behavioral action to re-establish a match and feeling of comfort. When a mismatch is detected from either external or internal sensory systems, a change in activity in the central and ANSs is produced. If the response is short-lived (one to three seconds), it is called arousal or an orienting reflex. If, however, the stimulus or event is recurrent, the brain eventually adapts and we habituate by updating the memories that serve as the reference. For example, people who live in a noisy city adapt to the ambient noise and eventually tune it out. Subsequent to this adaptation, it is only when they take a trip to the quiet countryside that the actual lack of noise seems strange and is quite noticeable. The mismatch between the familiar noisy background and the quiet setting leads to an arousal reaction that gets our attention. It is this departure from the familiar that gives rise to a signaling function that creates the experience of an emotion, alerting us to the current state of the mismatch.

In addition to the monitoring and control processes for regulation “in the here-and-now,” there are also appraisal processes that determine the degree of consistency or inconsistency between a current situation and the projected future. Appraisals of future outcomes can be broadly divided into optimistic and pessimistic (Pribram, 1970). Appraisals that project an inability to successfully deal with a situation may result in feelings of fear and anxiety. In keeping with the recent research on attentional bias (Olatunji et al., 2013), this appraisal might not be accurate, as it could be the result of hypersensitivity to cues that resemble past traumatic experiences in the current situation. Alternately, an inaccurate appraisal can be due to an instability in the neural systems, or a lack of experience or insight of how to effectively deal with the projected future situation (Pribram, 1970). Despite the lack of accuracy of the appraisal, the familiarity of the input can be sufficient to elicit a pessimistic response. This means we can easily get “stuck” in unhealthy emotional and behavioral patterns and that lasting improvements in emotional experience or behaviors cannot be sustained in the absence of establishing a new set point for the baseline. If behavior change or improved affective states are desired, it is therefore critical to focus on strategies that help to establish a new internal reference. As we successfully navigate new situations or challenges, the positive experience updates our internal reference. In essence, we mature through this process as we learn to more effectively self-regulate our emotions and deal with new situations and challenges. It is through this process that we are able to develop a new, healthier internal baseline reference against which we match inputs so that our assessments of benign inputs are more accurate and result in a feeling of safety and comfort rather than threat and anxiety.

## SELF-REGULATION AND STABILITY

Pribram and McGuinness (1975) and others have conducted numerous experiments that provide evidence that the higher brain centers that monitor the pattern-matching process can self-regulate by inhibiting or “gating” the information flowing into the brain. Where we focus our attention, for example, has a powerful effect on modulating inputs and thus on determining what gets processed at higher levels. In a noisy room filled with many conversations, for instance, we have the ability to tune out the noise and focus on a single conversation of interest. In a like manner, we can modulate pain from a stubbed toe or headache or desensitize ourselves to sensations like tickling, and self-direct our emotions (Pribram, 1971). Ultimately, when we achieve control through the process of self-regulation, it results in feelings of satisfaction and gratification. In contrast, failure to effectively self-regulate, and regain control often results in feelings of frustration, impatience, anxiety, overwhelm, hopelessness, or depression.

If the neural systems that maintain the baseline reference patterns are unstable, unsettled emotions, and atypical reactions are likely to be experienced. These neural systems can be destabilized by trauma, stress, anxiety or chemical stimulants, to name a few possibilities. Therefore, it is clear that responding in healthy and effective ways to ongoing inner and outer demands and circumstances, such as daily life situations, depends to a great extent on the synchronization, sensitivity, and stability of our physiological systems (McCraty et al., 2009b; McCraty and Childre, 2010).

Neural inputs originate from numerous organs, and muscles, especially the face. The heart and cardiovascular system, however, has far more afferent inputs than other organs and is the primary source of consistent dynamic rhythms (Cameron, 2002). In addition to afferent nerve activity associated with mechanical information such as pressure and rate that occurs with each heartbeat, continuous dynamically changing patterns of afferent activity related to chemical information is sent to the brain and other systems in the body. In terms of emotional experience, there are afferent pathways to the amygdala via the Nucleus of Tractus and the activity in the central nucleus of the amygdala is synchronized to the cardiac cycle (Zhang et al., 1986; Frysinger and Harper, 1990). Therefore, the afferent inputs from the cardiovascular system to the amygdala are important contributors in determining emotional experience and in establishing the set point to which the current inputs are compared.

In the context of this discussion, it is important to note that the heart’s rhythmic patterns and the patterns of afferent neurological signals change to a more ordered and stable pattern when one uses the heart-focused self-regulation techniques. Regular practice of these techniques, which include a shift of attentional focus to the center of the chest (heart area) accompanied by the conscious self-induction of a calm or positive emotional state, reinforces the association (pattern match) between a more coherent rhythm and a calm or positive emotion. Positive feelings then more automatically initiate an increase in cardiac coherence. Increased coherence initiated through heart-focused breathing tends to facilitate the felt experience of a positive emotion. Thus, practice facilitates the *repatterning process*.

This is important in situations where there has been a sustained exposure to truly high risk environments or trauma in the past, but that context is no longer in effect, and the patterns developed at that time no longer serve the individual in current safe environments.

Through this feed-forward process, regulatory capacity is increased and new reference patterns are established, which the system then strives to maintain, making it easier for people to maintain stability and self-directed control during daily activities, even during more challenging situations. Without a shift in the underlying baseline, it is exceedingly difficult to sustain behavioral change, placing people at risk of living their lives through the automatic filters of past familiar experience.

## SOCIAL COHERENCE

In social interactions, we also have set points or familiar habitual ways in which we perceive and respond. Consistent with the coherence model, social coherence is reflected in the harmonious quality of the network of relationships shared by individuals. In a socially coherent system, relationships are aligned in such a way as to allow for optimal collective function through efficient communication and shared energy resources (Bradley, 1987). If our familiar social set points reflect a pattern of harmony and support, then optimal social functioning in our interactions leads to an experience of safety, comfort, and well-being. On the whole, social coherence rests on the ability of group members to remain attuned to the group and the ability of the group to be organized and regulated according to mutually agreed upon norms (Bradley, 1987).

In most social contexts, individuals at times experience incoherent feelings towards one another, such as preconceptions or judgments, which are unspoken and can result in disruptions in optimal social interactions through miscommunication or other damaging social dynamics.

Studies in social incoherence indicate that in addition to generating unpleasant feelings and relational dynamics, physiological processes are engaged which have a direct bearing on our state of health. For example, studies of individuals in incoherent social situations, including social chaos or isolation, suggest that they are more susceptible to disease (Neser et al., 1971; Marmot and Syme, 1976; Berkman and Syme, 1979; Ornstein and Sobel, 1987; Hermes et al., 2009). Research conducted by James Lynch on social isolation indicates that the risks of isolation far exceed the combined risk for heart disease of smoking, obesity, lack of exercise, and excessive alcohol (Lynch, 2000). This is especially sobering and relevant for people suffering from trauma. As stated previously, aside from the experience of inner turmoil, one of the major symptoms of trauma is social alienation that often stems from depersonalization.

In contrast, the protective value of close, meaningful relationships has also become clear. Building upon studies that examine the role of social connection in disease (Cohen and Syme, 1985; Uchino et al., 1996; Ornish, 1998), James Coan and colleagues at the University of Virginia are investigating the role of social interactions and networks in wellness. Coan calls this Social Baseline Theory and suggests that the primary environments to which humans are adapted to are other humans and that the human brain implicitly assumes that it is embedded within a relatively predictable social network characterized by familiarity, joint attention, shared goals, and interdependence. In other words, social proximity is a “baseline” condition, and when proximity is maintained or reestablished, the brain is less vigilant for potential threats because it is familiar with the social environment. Thus, when we are in close proximity to our familiar social environment, and we have a match with our baseline state, we expend less emotional energy. We also expend less energy self-regulating. According to Social Baseline Theory, being alone is more effortful, because a variety of activities require more energy expenditures due to decreased load sharing and risk distribution (Beckes and Coan, 2011; Beckes et al., 2013; Maresh et al., 2013). Given the fragmented psychological state that predominates in trauma, one can speculate that for many, alienation has become the new norm, and even though isolation is innately stressful, a pattern of alienation under these circumstances can become habituated, such that social proximity might be experienced as a mismatch with the existing baseline and therefore add to the perceived stress burden rather than alleviate the burden. This can place the individual in a downward spiral due to a repetitive self-sustaining feedback loop of separation from one of the very resources that has been demonstrated to facilitate healing. This separation may be exacerbated by cultural patterns of marginalization in societies where those with perceived disabilities are shunned, judged and even blamed, sometimes by caregivers themselves (Thomas and Scharzbaum, 2011).

Reestablishing connectedness, whether inner or outer, such as in the case of trauma, is a complicated process, and yet it is one of the most important aspects that allows for reintegration. Levine (2010) speaks about the calming effect of social engagement and how the physiology of trauma compromises the capacity of an individual to be in the present moment and receive social support. Scaer (2012) speculates about the power of “face-to-face empathic attunement” between the client and therapist in a therapeutic setting to activate the limbic centers that are responsible for down-regulating the amygdala, which plays a central role in the physiology of trauma. Scaer notes that the skill of the therapist in establishing this container for reconnection to occur is of paramount importance. Therefore, it is critical for both client and therapist to be able to be present and engaged in an empathic exchange.

Research suggests that when individuals learn to sustain coherence while communicating with others, there is increased physiological linkage, and they become more sensitive to others so as to promote greater empathy and rapport, which allows for the process of heart felt connection to occur (McCraty, 2004).

## TRAUMA AND SELF-REGULATION

In considering the importance of reconnecting socially, as well as a few of the basic elements of trauma discussed previously, such as emotional dysregulation, intrusive memories, difficulty returning to homeostasis, inappropriate ANS arousal and reduced HRV, it becomes apparent that simple, straightforward techniques that effectively increase one’s self-regulatory capacity would be highly beneficial in bringing wholeness and harmony to not only one’s personal experience but also to one’s connectedness with others. Although the types and treatment of trauma are a highly complex topic involving many interrelated approaches and modalities, some central elements emerge in the literature. A review by Courtois and Ford (2013) of the principles of complex trauma and how it is expressed across individuals, suggests that crucial components for building a foundation of care includes emotional regulation, attention to wellness and stress management.

## SELF-REGULATION TECHNIQUES THAT INCREASE CARDIAC COHERENCE

The HeartMath self-regulation techniques and assistive technologies provide a systematic process for self-regulating thoughts, emotions and behaviors, and increasing physiological coherence (Childre and Martin, 1999; Childre and Rozman, 2003, 2005). Many of them are specifically designed to enable people to intervene in the moment they start to experience stress reactions or unproductive thoughts or emotions. Skill acquisition of the tools and techniques (Heart-Focused Breathing, Freeze Frame, Inner-Ease, Quick Coherence, Heart Lock-In, Prep, Shift and Reset, Getting In Sync, and Coherent Communication) are often supported by heart rhythm coherence feedback technology.

With practice, one is able to use one of the techniques to shift into a more coherent physiological state before, during and after challenging or adverse situations, thus optimizing mental clarity and emotional composure and stability. As previously discussed, in such a state, most people are able to more quickly find their “center,” gain new perspectives, and counter ineffective and maladaptive thoughts, feelings, and behaviors.

Effectively dealing with trauma and instating a new internal reference first involves increased self-awareness and recognizing triggers, reactions, and ongoing emotional undercurrents (fear, negative projection, insecurity, worry, etc.). Once one is more aware, the next step is learning how to consciously self-regulate and increasingly replace these feelings with more neutral or positive attitudes and perceptions.

The first step in most of the techniques is called Heart-Focused Breathing, which includes putting one’s attention in the center of the chest (area of the heart) and imagining the breath is flowing in and out of that area while breathing a little slower and deeper than usual. Conscious regulation of one’s respiration at a 10 seconds rhythm (0.1 Hz) increases cardiac coherence and starts the process of shifting into a more coherent state. In challenging situations or after a strong emotion has been triggered, Heart-Focused Breathing is often the step that most people can remember and find that it “helps take the intensity out” or “turn down the volume” of the reaction. As we have conscious control over breathing and can easily slow the rate and increase the depth of the breathing rhythm, we can take advantage of this physiological mechanism to modulate efferent vagal activity and thus the heart rhythm. This, in turn, increases vagal afferent nerve traffic and increases the coherence (stability) in the patterns of vagal afferent nerve traffic which influences the neural systems involved in regulating sympathetic outflow, informing emotional experience, and synchronizing neural structures underlying cognitive processes (McCraty et al., 2009b). While rhythmic breathing methods are an effective way to increase heart rhythm coherence, cognitively directed paced breathing is difficult for many people to maintain for more than about a minute before it becomes uncomfortable and distracting (Alabdulgader, 2012).

We have found that self-induced positive emotions can initiate a shift to increased cardiac coherence without any conscious intention to change the breathing rhythm (McCraty et al., 1995; Tiller et al., 1996). Typically, when people are able to self-activate a positive or calming feeling rather than remaining focused on their breathing, they enjoy the feeling shift and are able to sustain high levels of coherence for much longer time periods. However, people who are just learning the techniques or who experience strong emotional triggers may not be able to self-activate a calm or positive emotion. In these instances, using the Heart-Focused Breathing step can be used to start the process of regaining their composure and increasing their coherence. When their thoughts and emotions have slowed down and the intensity reduced, they can move to the next step of the various techniques, depending on the situation. Remembering to use any self-regulation approach requires effort, and ongoing mentoring or coaching can significantly help motivate clients to practice and sustain the use of the techniques (Bedell, 2010).

In addition to the techniques outlined above there are other approaches that also increase HRV coherence. For example, a study of Zen monks found that advanced monks tended to have coherent heart rhythms during their resting recording, while the ones that had been a monk for less than two years did not (Lehrer et al., 1999). A study of Autogenic Meditation also showed increased HRV coherence and found that cardiac coherence was strongly correlated with EEG alpha activity. The authors suggested that cardiac coherence could be a general marker for the meditative state (Kim et al., 2013a). However, this does not suggest that all meditation styles increase coherence, unless the coherence state is driven by a focus on breathing at a 10 seconds rhythm (Peng et al., 1999; Wu and Lo, 2008; Phongsuphap and Pongsupap, 2011) or a positive emotion. For example, a study examining HRV while reciting rosary or bead prayers and yoga mantras found that a coherent rhythm was produced by rhythmically breathing but not by random verbalization or breathing. The authors ascribed the mechanisms for this finding as due to changes in their breathing patterns to a six cycles per minute rhythm and concluded that the rhythm of mantras and rosary prayers were intentionally designed to induce coherent heart rhythms by individuals who had an intuitive understanding of the benefits of this rhythm (Bernardi et al., 2001). In a study of the effects of five different types of prayer on HRV, it was found that all types of prayer elicited increased cardiac coherence; however, prayers of gratefulness and prayers that focused on heart felt love resulted in definitively higher coherence levels (Stanley, 2009). There are also many studies showing that the practice of breathing at 6 breaths per minute, supported by HRV biofeedback, induces the coherence rhythm and has a wide range of benefits (Lehrer et al., 2003, 2006; Siepmann et al., 2008; Hallman et al., 2011; Henriques et al., 2011; Ratanasiripong et al., 2012; Beckham et al., 2013; Li et al., 2013). It has also been shown that tensing the large muscles in the legs in a rhythmical manner at a 10 seconds rhythm can induce a coherent heart rhythm (Lehrer et al., 2009).

## HEART RATE VARIABILITY COHERENCE FEEDBACK

In addition to clinical applications, HRV coherence training is often utilized to support self-regulation skill acquisition in educational, corporate, law enforcement and military settings. Several systems that assess the degree of coherence in the user’s heart rhythms are available. The majority of these systems, such as the emWavePro, or Inner Balance for iOS devices (HeartMath Inc), Relaxing Rhythms (Wild Divine), and Stress Resilience Training System (Ease Interactive), use a noninvasive earlobe or finger pulse sensor, display the user’s heart rhythm, and provide feedback on their level of coherence.

## CONCLUSION

The psychophysiological coherence model has informed the development of practical applications and approaches for increasing self-regulatory capacity and vagal tone in a wide range of populations, including individuals who have experienced trauma. Numerous studies have provided evidence that coherence training consisting of intentional activation of positive and calming emotions paired with HRV coherence feedback facilitates significant improvements in wellness and well-being indicators in a variety of populations.

The role of cardiac coherence in facilitating a resetting of adaptive response patterns through a shift in the physiological baseline reference to a healthier pattern appropriate for current contexts was highlighted as central to supporting the process of return to optimal function. While the experience of trauma is associated with a sense of fragmentation and loss of control that emerge from intrusive activations of the trauma stimulus, impairments in self-regulation, and difficulty returning to homeostasis, the practice of techniques that increase cardiac coherence is associated with an experience of intra and interpersonal synchronization, social harmony and wholeness. This is of particular relevance in circumstances where quality of life is significantly impaired, such as in the case of trauma. The process of re-patterning through intentional activation of positive emotions and generating an increasingly consistent state of psychophysiological coherence brings with it the possibility for addressing the primary defining components of the experience of trauma, thus allowing individuals to move out of the “stuck state” of dysregulation and fragmentation into a state of harmonious synchronized healthy function both at the individual and social levels.

## DISCLOSURES

Dr. Rollin McCraty is employed by the HeartMath Institute which is a non-profit research center supported by grants, donations, and some fee for service activities such as providing self-regulation trainings, sales of books, and heart rhythm coherence technologies, all is which is focused on services to education, service members, veterans, and non-profit social services agencies. The HeartMath Institute does not manufacture any devices, and if and when they are included in research projects or resold, are purchased from the manufacture in the same way as any other organization.

## Conflict of Interest Statement

The authors declare that the research was conducted in the absence of any commercial or financial relationships that could be construed as a potential conflict of interest.
